# The role of cytokines in modulating learning and memory and brain plasticity

**DOI:** 10.1177/2398212820979802

**Published:** 2020-12-18

**Authors:** Julie-Myrtille Bourgognon, Jonathan Cavanagh

**Affiliations:** Institute of Infection, Immunity & Inflammation, University of Glasgow, Glasgow, UK

**Keywords:** Cytokines, plasticity, inflammation, IL-6, IL-1β, TNF-α

## Abstract

Cytokines are proteins secreted in the central nervous system by neurons, microglia, astrocytes and infiltrating peripheral immune cells under physiological and pathological conditions. Over the last 20 years, a growing number of reports have investigated the effects of these molecules on brain plasticity. In this review, we describe how the key cytokines interleukin 1β, interleukin 6 and tumour necrosis factor α were found to support long-term plasticity and learning and memory processes in physiological conditions. In contrast, during inflammation where cytokines levels are elevated such as in models of brain injury or infection, depression or neurodegeneration, the effects of cytokines are mostly detrimental to memory mechanisms, associated behaviours and homeostatic plasticity.

## Introduction

Brain plasticity is the ability of the brain to change its activity and modify its connections throughout life in response to extrinsic or intrinsic stimuli. Indeed, synaptic plasticity supports learning and memory processes (Konorski, 1948) during which the strength and efficacy of the synaptic transmission change between neurons, accompanied by structural modifications of spines, dendrites and axons. We will describe the role of cytokines in plasticity such as long-term potentiation (LTP), a key process for memory formation involving *N*-methyl-d-aspartate receptors (NMDAR) and α-amino-3-hydroxy-5-methyl-4-isoxazolepropionic acid receptors (AMPAR; Bear, 1994), and synaptic scaling, an operation by which a neuron adjusts the postsynaptic strength of all its synapses in order to maintain normal neuronal network functions (Turrigiano, 2008). Neurons were, for a long time, thought to be the sole actors involved in plasticity but over the last decades, the roles of glial cells have switched from passive homeostatic elements to active modulators of synaptic plasticity and information processing. Among these glial cells are astrocytes that engulf synapses and surround blood vessels. Their many functions involve, among others, uptake and clearance of neurotransmitters such as glutamate and gamma aminobutyric acid (GABA; Sattler and Rothstein, 2006; Schousboe and Waagepetersen, 2005) and regulation of synaptic functions (Halassa and Haydon, 2010). Microglial cells also play a key role in modulating neuronal plasticity. They are a specialised population of tissue-resident macrophages broadly distributed in the brain parenchyma. Resting microglial cells are constantly scanning their environment by targeting synapses to monitor and regulate neuronal activity (Dissing-Olesen et al., 2014; Li et al., 2012). In response to infections, stress, degenerative diseases or any changes in the nervous system both activated astrocytes and microglia produce cytokines that modulate a large variety of physiological and pathological processes (Hanisch, 2002; Hanisch and Kettenmann, 2007; Sofroniew, 2013).

Cytokines are small pleiotropic signalling proteins classically secreted in response to pathogens or injury by cells of the immune system, including monocytes, macrophages, lymphocytes and vascular endothelial cells. This group of proteins comprises interleukins, chemokines, tumour necrosis factors, interferons, growth and cell stimulating factors and neurotrophins. In the brain, cytokines are constitutively expressed in various brain regions by activated glial and neuronal cells and are involved in several normal and pathological processes including sleep regulation (Krueger, 2008), neuronal development (Deverman and Patterson, 2009), alteration of the blood–brain barrier (Yarlagadda et al., 2009), modulation of neurotransmitter metabolism and synaptic plasticity (Miller et al., 2009; Raison et al., 2006). There is an increased interest for the role of cytokines in the brain due to the presence of inflammation in the brain in a wide range of diseases such as Alzheimer’s disease (AD), major disorder depression, epilepsy, stroke, amyotrophic lateral sclerosis and arthritis. In this review, we will discuss how cytokines modulate learning and memory and plasticity in the normal central nervous system (CNS) function and under pathological inflammatory conditions. Learning and memory experiments provide a significant amount of data as they are an effective way of integrating molecular and cellular plasticity mechanisms with systems-level and behavioural changes. We will focus mainly on interleukin 1β (IL-1β), interleukin 6 (IL-6) and tumour necrosis factor α (TNF-α) as they are the most studied cytokines in the brain so far.

### The role of cytokines (IL-1β, IL-6, TNF-α) on learning, memory and plasticity in physiological conditions

**Figure 1.**
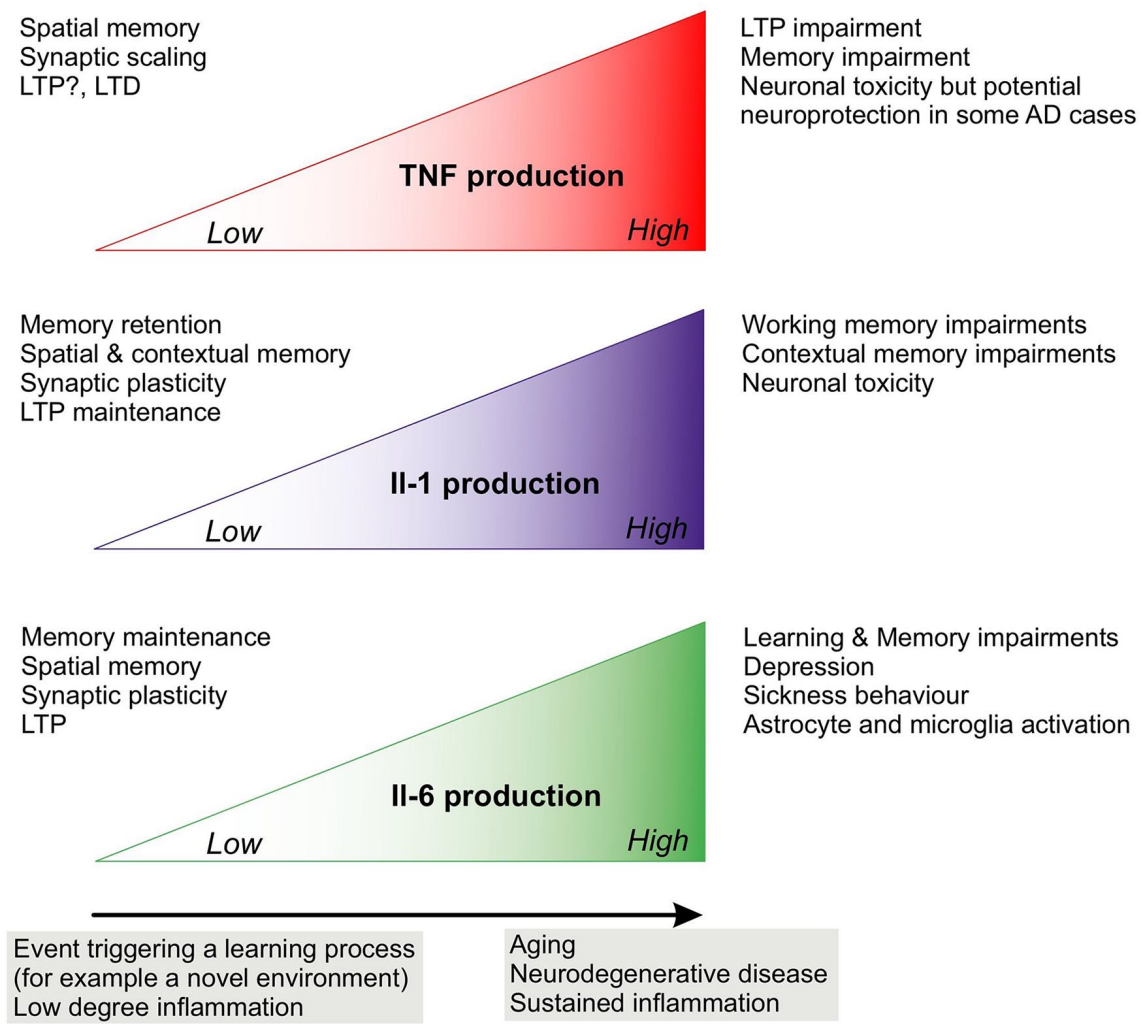
Summary of the effects of cytokines on learning, memory and plasticity.

**Figure 2.**
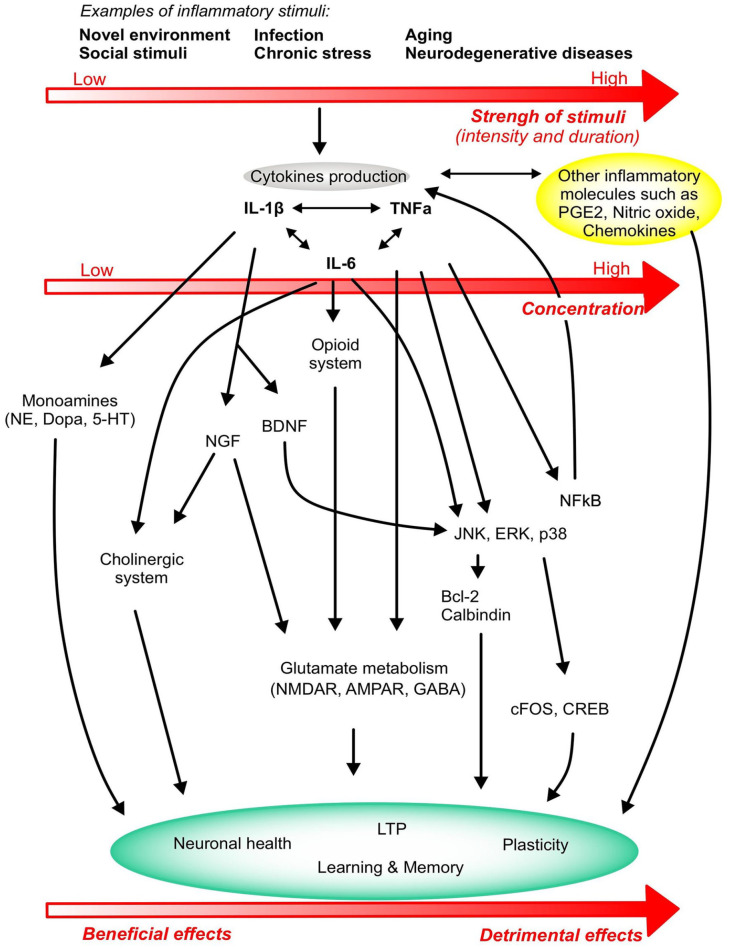
Overview of the major mechanisms of action of cytokines on plasticity and learning and memory. Stimuli of different intensity and duration activate the production of cytokines IL-1β, TNF-α and IL-6 that in turn modulate several metabolic and molecular pathways, ultimately affecting neurocircuits that regulate learning and memory function. The strength and duration of the stimulus determine the concentration and production levels of cytokines, leading the cytokine response to generate either beneficial effects on learning and memory or detrimental effects that ultimately progress towards neuronal death and cognitive deficits. Cytokine production also activates other inflammatory systems like PGE2, nitric oxide and other chemokines that will impact on the inflammatory status of the brain and the learning, memory and plasticity responses.

Cytokines and their receptors are expressed in the brain by neurons, microglia and astrocytes and their involvement in structural changes at the synaptic level was reported over 20 years ago (for review, Boulanger et al., 2001; Tonelli and Postolache, 2005). Periodic changes in synaptic transmission that underlie modification of behavioural states are often associated with adjustments in neurotransmitters at the synapse and those are influenced by cytokines (Miller et al., 2013). An overview of the effects of each cytokine on plasticity and learning and memory are summarised in Figure 1 and the mechanisms of action are presented in Figure 2.

#### IL-1β

Under normal resting conditions, IL-1β levels are usually below the level of detection and a stimulus is necessary to augment IL-1β expression. Many reports support the role of IL-1β in spatial recognition and contextual learning. IL-1β messenger RNA (mRNA) expression was found to be induced following fear conditioning and during spatial memory tasks (Depino et al., 2004; Goshen et al., 2007; Labrousse et al., 2009). No impairment was detected in IL-1 receptor (IL-1r) knockout (KO) mice for auditory cued memory nor visual memory (Avital et al., 2003) and intrahippocampal injection of IL-1β after training impaired the consolidation of contextual but not auditory fear conditioning memory (Gonzalez et al., 2009). However, mice depleted of any endogenous IL-1 receptor displayed hippocampus-dependent learning deficits (Avital et al., 2003), which highlight the complexity of IL-1 function in the brain (Table 1) and point to a possible role of IL-1β and IL-1α in normal memory function. A few studies have also shown that IL-1β is induced during short- and long-term plasticity and is necessary for LTP maintenance (Avital et al., 2003; del Rey et al., 2013). It is important to stress that IL-1 effects on memory are dose- and age-dependent (see Table 1): at low concentrations, injections of IL-1β appeared to promote synaptic plasticity and improved performance in passive avoidance conditioning whereas injections of higher IL-1β doses following a learning task resulted in impaired memory (Brennan et al., 2003; Goshen et al., 2007; Yirmiya et al., 2002). Most reports use ‘adult’ mice, usually between 2- and 4-month-old; however, an interesting study points out that the role of IL-1 might change when animals get older as only young (3 months) but not older mice (6 months) display deficits in spatial recognition (Takemiya et al., 2017). The authors suggest that synaptic sensitivity to IL-1β and that IL-1β function might evolve with age and with the appearance of age-related brain inflammation and elevation of proinflammatory molecules.

**Table 1.** Dose- and age-dependent effects of IL-1 on learning and memory.

References	Brain region	Animal model and compound concentrations	Age	Task	Effects
Goshen (2007)	Hippocampus	IL-1ra overexpression; IL-1ra 100 μg/mouse IL-1β low 1 ng or high 10 ng/mouse	Mice 2–4 months old	Contextual fear conditioning; water maze	Deficit
Brennan (2003); Yirmiya (2002)		Low-dose IL-1β 1–3 μg/kg; IL-1β 10 ng/rat	Brennan: rats 2-month-old Yirmiya: rats 6–8 months old	Passive avoidance	Improvement
Goshen (2007); Yirmiya (2002)		High-dose IL-1β 6–1000 μg/kg; IL-1ra 100 μg/rat	Yirmiya: rats 6–8 months old	Fear conditioning; passive avoidance	Deficit
Takemiya (2017)		IL-1ra knockout; IL-1β knockout;	Mice 3 and 6 months old	Water maze	Deficit for young mice
Avital (2003); Matousek (2010); Moore (2009)		IL-1 receptor type 1 knockout or IL-1β overexpression	Avita: mice 2–4 months old; others: adult mice	Contextual fear conditioning; water maze (spatial version)	Deficit
Avita (2003); Goshen (2007); Gonzalez (2009)	Amygdala; non- hippocampal-dependent tests	IL-1ra knockout; IL-1ra overexpression; IL-1ra 100 μg/mouse IL-1β low 1 ng or high 10 ng/mouse; IL-1β 5 ng/hippocampus	Mice 2–4 months old	Auditory cued fear conditioning; water maze (visual version)	No effect

IL: interleukin.

Peripheral IL-1β injection stimulates the release of monoamines in the brain (Song et al., 1999; Zalcman et al., 1994), which are known to modulate learning and memory (for review, Myhrer, 2003); for example, passive avoidance and acquisition of the water maze task were impaired in rats treated with a norepinephrine agonist (Sirviö et al., 1992); spatial memory performance was impaired in rats treated with 5-HT receptor agonist (Carli et al., 1992) and in rats having depleted dopamine (Whishaw and Dunnett, 1985). IL-1β can also modulate plasticity and memory by promoting the production of neurotrophic factors: nerve growth factor (NGF) stimulates acetylcholinesterase activity and facilitates LTP induction (Conner et al., 2009); brain-derived neurotrophic factor (BDNF) activates multiple signalling pathways involved in synaptic plasticity and memory formation such as TrkB–ERK pathway that leads to glutamate and GABA release, potentiation of NMDAR and upregulation of membrane AMPAR (reviewed in the study by Cunha et al., 2010).

#### IL-6

Rodent studies have shed light on the role of IL-6 in learning and memory (Figure 1) in different brain regions. IL-6 KO mice showed deficits in spatial learning tasks (Braida et al., 2004) and when IL-6 was blocked via the injection of anti-IL-6 antibodies in orbitofrontal cortex (OFC), rats had deficits in a reversal learning task (Donegan et al., 2014). IL-6 role in the striatum was investigated by Brennan et al; they showed that an intraperitoneal (IP) injection of IL-6 prior to an escape/avoidance task did not have any effect on the performance of the animal (Brennan et al., 2004). This task involves the dorsal striatal learning system and it is possible that IL-6 might not be required in that instance, or that the cytokine concentration was too low to have an effect. Several publications have also described a role of IL-6 in the hippocampus. Hippocampal-dependent avoidance learning performance was impaired when IL-6 was injected in the hippocampus (Ma and Zhu, 2000). Young and adult IL-6 KO mice perform better in a radial maze task compared to their wild-type (WT) counterparts (Braida et al., 2004); this difference was particularly noticeable in young mice, which suggest that the chronic lack of IL-6 facilitates learning and memory processes at a juvenile stage. IL-6 mRNA levels were upregulated after LTP induction in vitro and in freely moving rats, and blocking IL-6 led to prolonged LTP at the perforant path and improved memory in a Y-maze task (Balschun et al., 2004). Overall, these results suggest that IL-6 production and function in memory and plasticity might vary with age and brain region (Table 2).

**Table 2.** Effects of IL-6 on learning and memory.

References	Brain region	Mouse model	Task	Effects
Braida (2004)	Hippocampus	IL-6 knockout	Radial maze	Facilitatory effect
Heyser (1997)		IL-6 overexpressed (GFAP-IL-6 transgene)	Discriminated avoidance task	Deficit (increases with age)
Ma and Zhu (2000)		IL-6 infusion	Passive avoidance task	Deficit
Wu and Lin (2008)		IL-6 infusion	Forced swim task	Increased immobility
		IL-6 inhibitor	Forced swim task	Reduced immobility
	Amygdala	IL-6 infusion	Forced swim task	Increased immobility
		IL-6 inhibitor	Forced swim task	Reduced immobility
Donegan (2014)	Orbitofrontal cortex	Anti-IL-6 antibodies	Reversal learning (attentional set shifting test) after chronic intermittent cold (CIC) stress	Deficit
Donegan (2014)		IL-6 overexpression (AAV vector)	Reversal learning (attentional set shifting test) after chronic intermittent cold (CIC) stress	Deficit attenuated
Wu and Lin (2008)	Frontal cortex	IL-6 infusion or IL- 6 inhibitor	Forced swim task	No effect
Brennan (2004)	Dorsal striatum	IP injection of IL-6	Leverpress escape/avoidance task	No effect
Donegan (2014)		Anti-IL-6 antibodies	Reversal learning (attentional set shifting test) after chronic intermittent cold (CIC) stress	No effect
Roberts (2019)	Limbic and hypothalamic regions	IL-6 overexpression (GFAP-IL-6 transgene)	Open field, light/dark transfer, digging tests	Deficit
			Tail suspension test	No effect
			Forced swim task	Deficit

IL: interleukin; GFAP: glial fibrillary acidic protein; AAV: adeno-associated virus; IP: intraperitoneal.

The mechanisms by which IL-6 modulates cognitive function are far from being elucidated (Figure 2). Decreased ERK1/2 activation after IL-6 application correlates with impairments of LTP (Tancredi et al., 2000). A hypothesis is that the memory facilitation observed in mice lacking IL-6 could be due to the absence of inhibition from the endogenous opioid system as µ-opioid receptors were downregulated in IL-6 KO mice (Bianchi et al., 1999) and therefore unable to dampen learning and memory (Ukai et al., 2000; Ukai and Lin, 2002). IL-6 is also known to interact with the cholinergic system and peripheral injection of this cytokine reduced scopolamine-induced impairment of a passive avoidance task (Bertolucci et al., 1997). Moreover, intracellular calcium response to NMDA or glutamate was enhanced following chronic treatment of cultured cerebellar-granule neurons with IL-6 (Holliday et al., 1995; Qiu et al., 1995) meaning that IL-6 can also potentiate neurotransmitter responses and thus any dependent behaviour.

#### TNF-α

Very few studies have investigated TNF-α role under physiological conditions. Mice lacking TNF-α performed poorly in a novel object recognition task and in the Barnes maze test that evaluates spatial memory and learning effectiveness (Baune et al., 2008). A positive effect of TNF-α on learning and memory was also described in an escape/avoidance task where rats injected with TNF-α (6 μg/kg, intraperitoneal) made more avoidance responses and fewer escape responses compared to controls (Brennan et al., 2004).

TNF-α role in synaptic activity has been more extensively studied. TNF-α regulates synaptic scaling by promoting the insertion of AMPAR lacking GluR2 subunit at the plasma membrane of synapses, hereby promoting excitatory signalling over inhibitory signalling. TNF-α also modulates gliotransmission by elevating astrocytic calcium levels which in turn increase by twofold the number of glutamatergic exocytic vesicles. Released glutamate activates neuronal NMDAR at granule cells synapses of the dentate gyrus, hereby increasing excitatory synaptic activity and potentiation of GC synapses (Santello et al., 2011).

Beattie et al. showed, using neuronal cultures and acute hippocampal slices, that TNF-α was required for preservation of synaptic strength and increasing the number of AMPAR at the membrane, the number of synapses and the frequency of miniature excitatory post-synaptic currents (Beattie et al., 2002). Concomitant to these changes, TNF-α was found responsible for an increase in spine size of dendritic branches with recent spine loss, which constitute another synaptic scaling feature (Barnes et al., 2017). Other studies have confirmed that constitutive TNF-α is necessary for maintaining AMPAR at the synapse, and described how TNF-α also triggered GABAA receptors internalisation, highlighting the unique ability of this cytokine to modulate both excitatory and inhibitory plasticity (Stellwagen et al., 2005; Stück et al., 2012). Even though TNF-α can be produced by neurons, the above effects were generated by glial TNF-α in response to extracellular glutamate (Stellwagen and Malenka, 2006).

The role of TNF-α in other forms of plasticity is unclear. Some studies reported that LTP induction or maintenance did not require TNF-α as TNF-α KO mice displayed normal LTP in the CA1 pyramidal layer (Albensi and Mattson, 2000; Stellwagen and Malenka, 2006). However, a recent report highlighted a significant TNF-α-dependent inhibition of LTP in the stratum radiatum but not the stratum oriens of rats after either high-frequency priming stimulation or treatment with TNF-α (1.8 nM) via the phosphorylation of p38-MAPK, ERK and JNK (Singh et al., 2019), pointing to a differential regulation of LTP in the CA1 basal and apical dendrites. Findings regarding TNF-α and LTD are also conflicted. Although Albensi et al. showed that TNF-α is a key factor for the induction of hippocampal LTD via nuclear factor kappa B (NFκB) activation (Albensi and Mattson, 2000), that was not the case in other laboratories (Stellwagen and Malenka, 2006). Overall, these varying results could be explained by different experimental conditions such as theta burst or high-frequency stimulation, the area in which the stimulation and recordings are made, the use of different TNF-α concentration, time of incubation, cell culture or brain slices.

### The role of cytokines in plasticity, learning and memory under inflammatory conditions

Central and peripheral inflammation in response to pathogens, injury or disease is a defence mechanism by which microglia and astrocytes become activated and potentially produce chemoattractant proteins that promote extravasation of monocytes from the periphery into the brain (Ransohoff et al., 2003). Therefore, in the inflamed brain, cytokines are produced by microglia, astrocytes, neurons, peripheral inflammatory cells, endothelial cells, pericytes and choroid plexus; and cytokine receptors are present on neurons, astrocytes, microglia and vascular endothelial and perivascular cells (Kennedy and Silver, 2016; Konsman et al., 2006; Verma et al., 2006). TNF-α, IL-1β and IL-6 have the ability to stimulate each other’s production and can act synergistically (Donzis and Tronson, 2014) to modulate neuronal responses, plasticity and learning and memory (Figure 2), often translated by an elevated excitability of the brain and learning and memory changes that we describe below.

#### IL-1β

A detrimental role of IL-1β on learning and memory was described in an inflammatory environment (Figure 1) created by generating chronic elevated IL-1 levels in the brain. Spatial memory deficits were reported after chronic injection of IL-1β into the lateral ventricles of rats in an eight-arm radial maze (Taepavarapruk and Song, 2010). Mouse models of inflammation using chronic hippocampal overexpression of IL-1β for 2 weeks led to the appearance of inflammatory markers such as activated glia, elevated prostaglandin E2 (PGE2), increased hippocampal proinflammatory cytokine and chemokine mRNAs and lower levels of the plasticity-related gene Arc (Hein et al., 2010; Moore et al., 2009). These transgenic mice displayed delayed acquisition and decreased retention in the spatial water maze task, and impaired long-term contextual fear memory, while hippocampal-independent and short-term memory remained intact (Hein et al., 2010; Moore et al., 2009).

Models of peripheral infections have also been used to study cytokines in the brain. Peripheral infection can cause significant impairment of cognitive functions in individuals already suffering from neurodegenerative disease (Table 3; Perry et al., 2003) and to a lesser extent in healthy individuals (Dantzer et al., 2008). When injected intraperitoneally with *Escherichia coli*, IL-1β is upregulated in the aged rat hippocampus (Barrientos et al., 2009). The involvement of IL-1β in learning and memory was also studied in mice injected with *Legionella pneumophila* before subjecting them to the Morris water maze (MWM). Sick mice displayed impaired learning; their task performance was, however, restored to control levels if they were treated with an anti-IL-1β antibody (Gibertini et al., 1995). It is worth noticing that the effects of IL-1β depend (1) on the intensity of the protocol, as a high frequency of training sessions were more likely to highlight an IL-1β–related memory impairment in rodents than when spaced protocols were used (Gibertini, 1998) and (2) on the concentration of IL-1β that is injected in the animal, with low dose (100 ng/mouse) leading to learning impairment and high dose of IL-1β (1000 ng/mouse) facilitating spatial navigation learning (Gibertini, 1998). Lipopolysaccharide (LPS) is another compound regularly used to study the effects of cytokines during an infection and it classically generates sickness behaviour-related symptoms (Dantzer, 2001; Goshen et al., 2008; Kent et al., 1992; Larson and Dunn, 2001; Maier and Watkins, 1998). Following LPS injection in mice lacking caspase-1, the enzyme that cleaves the inactive pro-IL-1 into its active form, mice displayed reduced sickness behaviours and decreased levels of proinflammatory markers such as PGE2 (Mastronardi et al., 2007). IP injection of LPS increased IL-1β protein levels in the cortex, hippocampus and hypothalamus of rat (Nguyen et al., 1998) and impaired contextual but not auditory fear conditioning (Nguyen et al., 1998; Pugh et al., 1998), an effect that was abolished by subcutaneous injection of the endogenous antagonist IL-1ra (Pugh et al., 1998). A mouse model of septic encephalopathy with blood–brain barrier disruption was characterised by astrogliosis and upregulation of hippocampal IL-1β and its receptor IL-1R1, and altered LTP was recovered when hippocampal slices were pre-incubated with an IL-1R1 antagonist (Imamura et al., 2011). IL-1β impairment of LTP was recorded in the Schaffer collateral–CA1 synapses and in the associational/commissural fibre–CA3 synapses; LTP deficit was found to be dependent on the activation of mitogen-activated protein kinases (MAPKs) and the presence of NMDAR (Hoshino et al., 2017; Kaminska et al., 2009). Interestingly, LTP was not impaired at mossy fibre–CA3 synapses, possibly because these synapses are NMDAR-independent (Nicoll and Schmitz, 2005).

**Table 3.** Effects of cytokines on patient’s cognition in different conditions.

References	Condition	Cytokine	Effects
Perry et al., (2003)	Peripheral infection added to neurodegenerative disease	IL-1β	Cognitive impairment
Dantzer (2008)	Peripheral infection in healthy individuals	IL-1β	Cognitive impairment
Weaver (2002), Economos (2013), Elderkin-Thompson et al. (2012), Foster (2002), Palotás (2002)	Chronic inflammatory diseases; ageing	IL-6	Sickness and depressive behaviours; Cognitive decline
Cohen (2003)	LPS injection	IL-6	Declarative and working memory impairments
Mao (2016), Mutso (2012)	Chronic pain	TNF-α	Memory deficits, reduced hippocampal volume

IL: interleukin; LPS: lipopolysaccharide; TNF: tumour necrosis factor.

Stressors like inescapable shocks also trigger an increase of IL-1β protein levels in the brain, although this increase can be difficult to highlight as it is likely to be masked by an inhibitory action of glucocorticoids (Nguyen et al., 1998). Chronic mild stress triggered an increase of hippocampal IL-1β protein levels and depression-like symptoms in rodents, but mice lacking the IL-1 receptor or with an astrocytic overexpression of an IL-1 antagonist did not display stress-induced behavioural nor neuroendocrine changes (Goshen et al., 2008). Social isolation also elevated Il-1β protein levels in the hippocampus and the cerebral cortex and consequently led to contextual fear-conditioning impairment (Pugh et al., 1999). Similar to isolation studies, rats infused with IL-1β in the hippocampus displayed contextual but not auditory deficits after fear conditioning, and this effect was prevented by intracerebral injection of IL-1ra (Pugh et al., 1999). IL-1 was also found to impair working memory using a three-panel runway task (Matsumoto et al., 2001, 2004). In a rodent model of seizure where the mouse cecum is ligated then punctured, IL-1β mRNA expression in the hippocampus was upregulated, escape latency in the MWM was impaired and LTP decreased; the use of an IL-1 antagonist rescued LTP and learning and memory abilities (Han et al., 2016). High hippocampal IL-1β levels found in aged or stressed rats was also associated with impaired LTP (Murray and Lynch, 1998). Under stress conditions, it is likely that microglial IL-1β production increase is linked to the activation of the purinergic receptor P2R×7 (Aricioglu et al., 2019), which is a key protein that supports stress and depression mechanisms (Ribeiro et al., 2019).

In a model of Parkinson’s disease, elevated IL-1β production in the brain worsened neurodegeneration course by supporting neuronal death, either directly or via nitric oxide production (Pott Godoy et al., 2008). IL-1β levels are elevated in AD brain as IL-1β was produced in response to Aβ exposure in cell culture (Meda et al., 1999) and was shown to contribute to cognitive deficits, tau phosphorylation and Aβ pathology in aged 3× Tg-AD mice (Kitazawa et al., 2011). Aβ-induced astrocytic IL-1β is also suspected of supporting the overexpression and activity of acetylcholinesterase hence exacerbating the cholinergic dysfunction linked to cognitive impairment observed in AD (Li et al., 2000).

Some mechanisms by which IL-1β modulates memory function have been described. It has been reported that the synaptic sensitivity to IL-1β increases with age due to a ratio change of the IL-1-receptor type 1 subunits AcP (proinflammatory) and AcPb (prosurvival) that facilitates memory impairment (Prieto et al., 2015). There is evidence also for the arachidonic cascade and the glutamatergic system to be influenced by IL-1β. Bilateral injection of IL-1β in the hippocampus of rats increased cyclooxygenase-2 (COX2) protein levels and activated the arachidonic cascade and the release of PGE2 in the hippocampus (Matsumoto et al., 2004; Molina-Holgado et al., 2000). Hippocampal PGE2 injection led to more working memory errors than in control group (Matsumoto et al., 2004). Similarly, selective and competitive NMDAR antagonists caused deficits in working memory performance in the three-panel runway task (Ohno et al., 1992). An NMDAR agonist concurrently administered with IL-1β into the hippocampus reversed the working memory impairment induced by the cytokine. IL-1β also modulates calcium influx through NMDARs (Viviani et al., 2003). Moreover, elevated IL-1β protein levels in the brain could inhibit LTP by interfering with BDNF signal transduction and dendritic spine morphology (Tong et al., 2012).

#### IL-6

In illnesses such as depression, arthritis and others where chronic inflammation is settled, patients report sickness and depressive behaviours and cognitive decline, often accompanied by elevated levels of IL-6 (Weaver et al., 2002). Individuals given a dose of LPS did not report feeling sick but performed poorly during declarative memory tests and better in working memory tasks (Cohen et al., 2003). A lot of rodent studies have confirmed these detrimental effects of IL-6 on cognition and behaviour. Mice lacking IL-6 displayed reduced sickness behaviour and depressive-like social behaviour induced by systemic and central injections of IL-1β and LPS was lower in IL-6−/− mice than in their WT counterparts (Bluthé et al., 2000). Similarly, turpentine-induced abscess or influenza infection did not cause any behavioural deficits in IL-6−/− mice (Kozak et al., 1997). Sparkman et al. evaluated the ability of these animals to integrate new information after the acquisition period of an MWM task by injecting LPS. Contrary to WT mice, IL-6 KO animals failed to display any working memory impairment and lacked the expected LPS-induced increase in TNF-α and IL-1β mRNA in hippocampal neurons but not in the periphery (Sparkman et al., 2006). However, LPS injection still activated a marker of neuronal activity c-Fos in neurons of the nucleus tractus solitarius that receive peripheral information via the vagal nerve (Sparkman et al., 2006; Vallières and Rivest, 1999). These data support the fact that IL-6 is not fundamental for periphery-to-brain communication but is required for LPS-induced production of TNF-α and IL-1β in the brain and for the expression of some behavioural impairments. Mice lacking or expressing IL-6 have also been used to highlight the role of IL-6 in the limbic system in relation to anxiety-like and depressive-like behaviours. IL-6 KO mice showed alterations in such behaviours (Chourbaji et al., 2006; Wu and Lin, 2008). Decreased exploratory behaviour in a light–dark transfer test and digging behaviours were reported in mice overexpressing IL-6 together with a longer time spent immobile in a forced-swim test (Roberts et al., 2019; Table 2) as a readout of the ability of the animal to adapt upon exposure to an inescapable stressor (Molendijk and de Kloet, 2019).

IL-6 plays a key role in activating astrocytes and microglia during inflammatory conditions. IL-6 KO mice displayed reduced reactive astrogliosis and microgliosis after IP injection of the toxic glutamate receptor agonist kainic acid (model of seizure; Penkowa et al., 2001) or subcutaneous injection of the neurotoxicant 1-methyl-4-phenyl-1,2,3,6-tetrahydropyridine (model of selective dopaminergic cell injury, which occurs in Parkinson’s disease; Cardenas and Bolin, 2003). Indeed, IL-6 KO mice had a reduced astrocytic population and a decreased ability to activate microglia (Klein et al., 1997); and in a murine model of experimental autoimmune encephalomyelitis, IL-6 blocked the astrocytic response to interferon-γ and the release of microglial inhibitory molecules such as galectin-1 and HO-1 (Savarin et al., 2015). This glial cell activation by IL-6 is likely to happen in ageing and neurodegenerative diseases. Studies have described the elevation of IL-6 in the plasma of elderly individuals and its correlation with cognitive decline, with females being more sensitive to higher IL-6 levels (Table 3; Economos et al., 2013; Elderkin-Thompson et al., 2012; Weaver et al., 2002). These results were confirmed in a mouse model of accelerated ageing in which IL-6 protein levels were increased by 50% and 30% in the hippocampus and cortex of aged mice, respectively, compared to controls (Tha et al., 2000). Similarly, IL-6 gene and protein expression in glial cells were found to be upregulated in the cerebellum, cortex and hippocampus of mice as they age naturally (Ye and Johnson, 1999). IL-6 detrimental effects on cognition could be linked to neurodegeneration and calcium homeostasis which is perturbed in the brain of Alzheimer’s patients and aged individuals (Foster and Kumar, 2002; Palotás et al., 2002), with the expression of a key calcium-binding and buffering protein calbindin being reduced in rodent models of dementia (Heyser et al., 1997; Palop et al., 2003; Figure 2). Mice chronically expressing astrocytic IL-6 were found to express a progressive age-related decline in avoidance learning performance that correlated with pre-synaptic loss, typically associated with behavioural deficits (Cunningham et al., 2003) and a decrease in cortical and hippocampal neuronal calbindin that plays a protective role in AD (Kook et al., 2014). However, in the early phase of AD, IL-6 participates in a reduction in Aβ deposition as IL-6 overexpression in a mouse model of AD led to enhanced microglia activation and phagocytosis activity (Chakrabarty et al., 2010).

#### TNF-α

The role of TNF-α in learning and memory under inflammatory conditions is likely to be age dependent. Indeed, studies using 30-day-old mice overexpressing TNF-α did not find any learning and memory impairment in the water maze test (Aloe et al., 1999; Fiore et al., 1996), whereas older mice showed impaired memory in a passive avoidance task (Fiore et al., 1996), in the water maze (Bjugstad et al., 1998) and in a three-panel runway task (Matsumoto et al., 2002). Adult rats chronically overexpressing five times the normal levels of murine neuronal TNF-α also display spatial memory impairments in a water maze paradigm (Pettigrew et al., 2008, 2016). In these animals, hippocampal synaptic plasticity measured by LTP and paired-pulse facilitation are comparable to those measured in the immature hippocampus of neonates, which support a role of TNF-α in maturation of the synaptic network (Pettigrew et al., 2008, 2016). Another report points to a beneficial role of TNF-α in memory recovery as mice lacking TNF-α that have recovered from pneumococcal meningitis displayed impaired water maze performance compared to WT controls (Gerber et al., 2004).

Chronic pain is often accompanied by memory deficits and a reduced hippocampal volume (Table 3; Mao et al., 2016; Mutso et al., 2012). In a model of chronic pain, a pared sciatic nerve leads to microglia activation, increases TNF-α levels and impairs LTP in the hippocampus, reduces the density of presynaptic boutons, as well as the functional synaptic connectivity and BDNF expression in CA1 neurons (Liu et al., 2017; Ren et al., 2011). These synaptic changes are TNF-α-dependent as similar results were obtained when TNF-α was injected in the brain, but not when the experiment was repeated with mice lacking the TNF-α receptor TNFR1 (Liu et al., 2017; Ren et al., 2011).

Astrocytic and microglial TNF-α release under inflammatory conditions is a feature of neurodegenerative diseases (Chung and Benveniste, 1990; Rojo et al., 2008; Yin et al., 2017) and is associated with increased levels of Aβ, tau and neuronal cell death (Janelsins et al., 2008; McAlpine et al., 2009). CSF levels of TNF-α, produced by brain cells, are increased in some AD patients (Tarkowski et al., 1999); TNF-α mediates LTP inhibition as well as memory deficits caused by Aβ (Rowan et al., 2007; Tobinick, 2009; Wang et al., 2005). In aged APP/PS1 mice, a model of AD in which TNF-α levels are twofold higher than in their WT counterparts, there is a marked TNF-α-dependent inhibition of LTP possibly due to elevated TNF-α production by Aβ-dependent glial cell activation (Singh et al., 2019). Several other studies have reported that in mice and hippocampal slices, TNF-α abolished LTP and had deleterious effects on working memory (Cunningham et al., 1996; Curran and O’Connor, 2003; Pickering and O’Connor, 2007; Ren et al., 2011; Tancredi et al., 1992; Wall et al., 2015), while cognitive deficit is reduced by pharmacological inhibition of TNF-α in mice (Shin et al., 2014). Interestingly, earlier in vitro studies have reported a protective role of TNF-α in AD, with human TNF-α levels inversely correlated with levels of a marker of neuronal degradation tau and in vitro data linking TNF-α incubation of neuronal cells to an increase in bcl-2, which can downregulate apoptosis (Tarkowski et al., 1999). TNF-α has also been shown to protect against glutamate, free radical and Aβ toxicity in enriched cultures of primary neurons (Barger et al., 1995).

In an inflammatory context, TNF-α levels can rise from picomolar to millimolar levels; phosphorylation of MAPKs (Figure 2) have been described during TNF-α inhibition of LTP in the molecular layer of the dentate gyrus and hippocampal apical dendrites (Butler et al., 2004; Singh et al., 2019). Elevated TNF-α concentration can also activate NFκB and potentiate NMDAR and AMPAR via TNFR1 activation, blocking glutamate transporter activity and promoting glutamate neurotoxicity (Bernardino et al., 2005; Zou and Crews, 2005), leading eventually to neuronal and oligodendrocyte cell death (Akassoglou et al., 1998; Li et al., 2004). Moreover, TNF-α favours synaptic transfer of calcium-permeable AMPAR lacking GluR2 subunit, hence potentially increasing the risk of neuronal toxicity (Stellwagen et al., 2005).

Surprisingly, the effects described above do not occur in the dorsolateral striatum of rodents. In this region rich in inhibitory GABAergic neurons, TNF-α promotes the internalisation of AMPAR permeable to calcium thereby reducing synaptic strength, which could be an adaptative mechanism of the brain to delay motor symptoms in diseases characterised by movement disorders and elevated TNF-α levels (Lewitus et al., 2014). Overall TNF-α roles on plasticity depend on the anatomical location of TNF-α action and on the level of expression of TNF-α (Butler et al., 2004; Cunningham et al., 1996; Tancredi et al., 1992; Wall et al., 2015).

## Conclusion

Cytokines are much more than basic signalling inflammatory molecules as they have key roles in modulating cognitive functions in physiological conditions. We propose that the length, strength and location in a particular area of the brain of cytokine activity will dictate the activation of specific signalling pathways together with the stimulation of other inflammatory systems that will contribute to the complexity of the response. This renders the distinction between mechanisms that would be solely engaged either in physiological or in inflammatory conditions very difficult. We suggest that the homeostasis equilibrium shifts when pathological, chronic expression of cytokines sustains in time; then the cytokine actions often become detrimental and lead to neuronal death and learning and memory impairments. In that view, a better understanding of the interaction between the whole immune system and synaptic plasticity would certainly be key to develop treatments for neuronal, cognitive and mood disorders that present deficits in these mechanisms.
